# Psychiatric in-patients who are parents: what interventions are tailored to their needs and how do they experience care? A systematic review and data synthesis

**DOI:** 10.1192/bjo.2023.67

**Published:** 2023-06-22

**Authors:** Abigail Dunn, Hanna Christiansen, Chloe Elsby-Pearson, Jaqueline Kramer, Eliza Swinburn, Belinda Platt, Sam Cartwright-Hatton

**Affiliations:** University of Sussex, Falmer, UK; Philipps-Universität, Marburg, Germany; LMU University Hospital, Munich, Germany

**Keywords:** In-patient treatment, patients, psychiatric nursing, psychosocial interventions, social functioning

## Abstract

**Background:**

Little is known about the experiences of parents who are in receipt of in-patient psychiatric care or about what interventions are employed to support them in their parenting role.

**Aims:**

The objective of the current study is to review two complementary areas of research: (a) research examining interventions developed to support the parent–child relationship within these settings; and (b) research focused on the experience of parents in in-patient settings.

**Method:**

For studies reporting on parents’ experience, qualitative accounts of past or present psychiatric in-patients (child aged 1–18 years) were included. For intervention studies, the intervention had to focus on supporting the parenting role and/or the parent–child dyad of parents (child aged 1–18 years) in current receipt of in-patient care. Four bibliographic databases (PubMed, SCOPOS, Web of Science and PsychINFO) were searched for relevant published and unpublished literature from 1 January 1980 to 26 July 2022. Intervention studies were appraised using the Mixed Methods Appraisal Tool. Qualitative papers were assessed using the Critical Appraisal Skills Programme tool. Data were extracted using tools designed for the study. Qualitative data were synthesised using thematic analysis. The protocol was registered with the International Prospective Register of Systematic Reviews (reference CRD42022309065).

**Results:**

Twenty-four papers (eight intervention studies and 16 studies examining parent experience) were included in the review. In-patient parents commonly reported hospital admission as having a negative impact on their parenting. Very few robust reports of interventions designed to support parents in receipt of psychiatric in-patient care were found.

**Conclusions:**

Despite the identified need for support by parents who are receiving in-patient care, there is currently no intervention of this nature running in the UK health service.

Of the approximately 16 500 psychiatric in-patients in the UK at any time, around one-quarter are parents of dependent children, with similar figures reported internationally.^1,2^ Parents in in-patient care are not a homogenous group; however, for the majority, hospital admission requires separation from their children. In many cases, this follows a period of acute mental illness and sometimes a difficult or non-voluntary admission process, which is distressing to parent and child.

For most adults with children, parenthood is an integral part of their identity, bringing both reward and challenge. This is no different for parents with serious mental health problems.^3^ However, this parenting role is rarely acknowledged by services.^4^ Adults experiencing serious mental illness can provide nurturing parenting and derive satisfaction from this.^5,6^ However, the challenges of parenting are understandably greater for those with severe mental illness. The ability to provide appropriate care may be compromised by symptoms and treatment, behavioural and relational challenges, financial hardship and isolation from the networks that parents call upon.^7^ Furthermore, mental health difficulties are often associated with specific parenting attributes, such as challenges containing children's emotions, boundary setting and overprotection.^8,9^ Consequently, having a parent with severe mental illness is sometimes associated with impaired psychosocial outcomes for children.^10^

Although there has been limited research focusing on outcomes for children whose parents are in-patients in psychiatric units, there is evidence that such children are at risk of adverse outcomes, including being re-housed, (e.g. into foster care), poor school readiness and abuse.^11,12^ Such parents also report increased psychological and behavioural problems in their children.^13^

Parents who have received in-patient care largely characterise their experience as negative: hospital admission is seen as having a detrimental impact on the parenting role.^14^ For some, this rupture may continue beyond discharge, owing to child removal or because the relationship is perceived to be irretrievably damaged. Such parents also report low confidence in parenting and considerable parenting challenges.^15^ Children report profound disruption to their lives, including negative experiences when visiting parents in hospital.^16^

However, negative outcomes are not inevitable, with many parents providing excellent care to their children as they manage their mental health and some children reporting positive aspects to parental mental ill health.^17^

The provision of parenting support to parents with mental health difficulties (regardless of whether they are in-patients) is rare but likely to have cascading benefits for the family, including reducing the intergenerational transmission of mental health difficulties. A growing body of research suggests that effective interventions can be delivered to parents who experience a range of diagnoses.^18,19^ Furthermore, the literature suggests that parents overwhelmingly want support in their parenting role, want this support to occur preventively rather than in response to crisis and want it to exist beyond the perinatal period.^20^

Despite the challenges faced by parents in in-patient psychiatric care and the corollary risks to their children, there have been limited efforts to develop an evidence base of interventions for this vulnerable group. In a systematic review of interventions to support parents with severe mental illness, only two of 18 studies were delivered to parents during in-patient or residential treatment, with one delivered post-discharge.^21^ Of the in-patient studies, one comprised a case-note review of co-admitted parents and children, with no reported change statistics.^22^ The second focused on mothers with comorbid substance misuse and mental illness, with limited information about the intervention or outcomes.^23^ The third, delivered as post-discharge home visits for mothers with psychosis, focused on minimising readmission and did not specifically engage with parenting.^24^

There is a similar lack of research exploring the experience of being admitted to psychiatric care as a parent: a recent review of the experiences of in-patients included no mention of parenthood. An earlier review, which focused on support needs of families when a parent is admitted to hospital, included just six papers (of 18) that focused on the specific experience of parents.^25^

The current review extends the evidence base on support for parents using psychiatric in-patient care in two complementary areas of research:

- interventions to support the parent–child relationship within these settings;
- the experience of parents in in-patient settings.

## Method

This systematic review is reported in line with PRISMA guidelines.^26^ This paper is based on a doctoral thesis (PhD) by A.D.^27^

### Protocol registration

The protocol was registered the International Prospective Register of Systematic Reviews (reference CRD42022309065).

### Ethical statement

The authors have abided by the Ethical Principles of Psychologists and the Code of Conduct as set out by the British Association for Behavioural and Cognitive Psychotherapies (BABCP) and the British Psychological Society (BPS).

### Eligibility

Broad inclusion criteria were applied (Table 1). Papers were considered if they included primary research published in any country between January 1980 and July 2022. All designs were eligible.

**Table 1:** PICOs schema used to inform eligibility criteria

Intervention studies		Qualitative studies	
Patient, problem or population	Parent accessing in-patient care for mental health treatment with child aged 12 months to 18 years	Patient, problem or population	Parent accessing/accessed in-patient care for mental health treatment with child aged 12 months to 18 years
Intervention	Parenting intervention targeting parent or parent–child dyad	Interest	Experience, views, opinions
Comparison	None	Context	Mental health in-patient care
Outcome	Any outcome for parent OR child (other than compliance with the intervention).		
Setting	In-patient psychiatric care	Setting	

For studies reporting parents’ experience of being psychiatric in-patients, past or present psychiatric in-patients with a child aged 12 months to 18 years at the time of treatment were included. Where both in-patients and community patients were included, data were extracted only for in-patients. For intervention studies, interventions had to be focused on supporting the parenting role and or the parent–child dyad, where the child was aged 12 months to 18 years, and the parent was in current psychiatric in-patient care.

Papers were excluded if they had no English-language abstract and/or the full paper was unavailable in English or German.

### Information sources

PubMed, SCOPOS, Web of Science and PsychINFO were searched for papers from 1 January 1980 to 4 December 2021 with an extension to cover 4 December 2021 to 26 July 2022.

### Search terms

The search terms were: Parent*OR mother*OR father*AND inpatient*OR ‘mental health unit*’OR‘psychiatric unit*’OR ‘psychiatric ward*’OR‘psychiatric hospital*’OR‘mental health rehabilitation unit'OR‘mental health residen*’OR‘mental health hospital*’. Reference lists of prior reviews and of final included papers were searched. Relevant academics were asked to identify additional papers and/or unpublished materials.

### Study selection

**Fig. 1:**
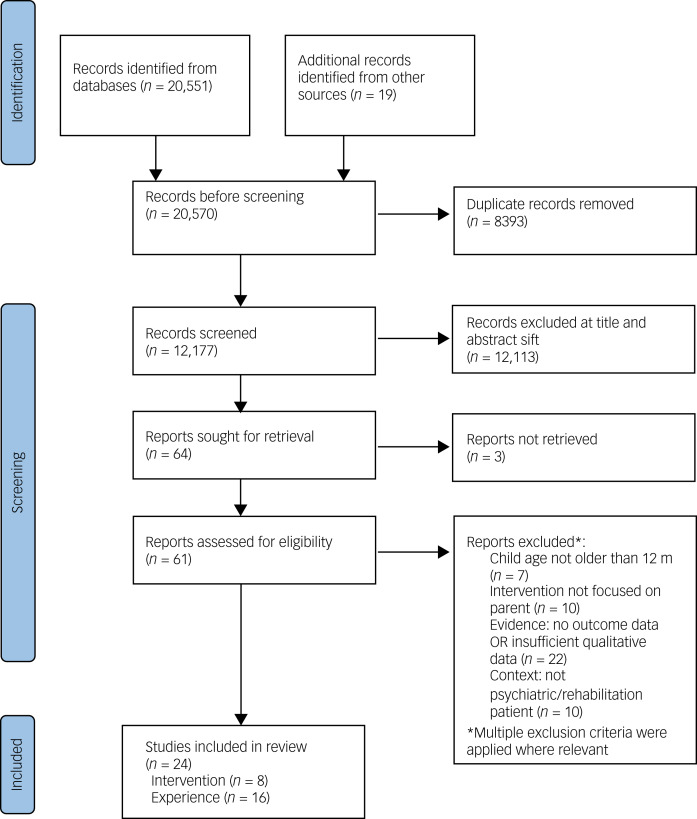
Flow diagram of studies included in the results.

Following de-duplication, two reviewers from a pool of six independently screened 20% of titles and/or abstracts against the inclusion criteria. Disagreement was below 1% and was resolved via discussion. The remaining were screened by a single reviewer. See Fig. 1.

Where possible, full-text papers were obtained for all studies that were retained following title/abstract screening. These were independently screened by two reviewers from a pool of three, with discrepancies resolved in consultation with a fourth. German-language papers were doubled-coded by two fluent German speakers, with additional discussion with the first author to reach consensus. For 61 full-text papers, there was a concordance rate of 95%. Three papers were resolved by discussion.

### Data extraction

Data were extracted using either a qualitatively or a quantitively orientated data-extraction form (included in the Supplementary materials available at https://doi.org/10.1192/bjo.2023.67) generated by the team. All papers were extracted twice (by two members of a pool of six), with discrepancies resolved collaboratively.

### Methodological quality

Methodological quality was assessed following data extraction, to evaluate the risk of bias. No papers were removed as a result. All papers were assessed by two coders (from a pool of four). For four of 26 papers where ratings were not in perfect agreement, ratings were resolved via discussion.

Intervention studies were appraised by two reviewers (from a pool of four) using the Mixed Methods Appraisal Tool (MMAT) 2018, which comprises two screening questions and five criteria focused on the paper type (i.e. randomised controlled trial (RCT) or descriptive study) using three response categories (Yes = 2; No = 0; Can't Tell = 0).

Qualitative papers (exploring experiences of parents) were assessed for quality using the 2018 Critical Appraisal Skills Programme (CASP) tool for qualitative research by two raters. This ten-item checklist uses three response categories (No = 0; Can't Tell = 0; Yes = 2). To increase sensitivity, an additional response category (Somewhat = 1) was included.^28^ See Tables 2 and 4.

**Table 2:** Description of included quantitative papers

Reference	Intervention details: elements focused on support for parenting or parent–child dyad (duration)	Study type	Population	*N* (control)	Follow-up	Outcomes	Intervention effects	Quality assessment (MMAT)
Besier (2011) Germany	Co-admission + parent–child interaction interventions (e.g. massage). (3 weeks with 1 week extension)	Pre–pre–post–post	Parents admitted for in-patient treatment. Diagnoses: neurasthenia and depression. Child ages: 0–2 years (*n* = 48); 1–6 years (*n* = 141); 7–11 years (*n* = 168); 12–17 years (*n* = 39).	256	4 weeks before admission, pre-admission, post-admission, 3 months post-discharge	Parent: psychiatric symptoms, quality of life. Child: behavioural screen, quality of life	Significant improvement in parental mental health and quality of life with both maintained at follow-up. Significant improvement in child behaviour which was maintained at follow-up.	4
Fritz (2017) Germany	Intervention: co-admission + SEEK: six-module group programme: three modules from the parent's perspective – psychoeducation around mental illness and coping strategies, dealing with stress and warning signs; three from child's perspective – psychoeducation on mental health reciprocity, child basic needs and mental health (5 weeks).Control: co-admission	Pre–post	Parent and children both with mental health diagnosis. Parental diagnoses: schizophrenia/schizoaffective and depressive disorder. Child ages: 0–6 years	25 (26)		Parent: psychiatric symptoms, parental stress. Child behaviour	Intervention: significant reduction in parental strain and symptoms. Control: significant reduction in parental stress and symptoms	4
Fritz (2018) Germany	See above	Pre–post–post	Parent and child both with mental health diagnosis. Parental diagnoses: schizophrenia/schizoaffective, affective disorder, depressive disorder, bipolar. Child ages: 0–6 years.	28 (26)	6 months post-discharge	See above	Intervention: significant reduction in overall psychiatric symptoms, depression and anxiety. Significant reduction in parental stress. All effects maintained at follow-up. Control: significant reduction in overall psychiatric symptoms and in depression. Significant reduction in parental stress. Reduction in depression and stress maintained at follow-up	4
Healy (1993) UK	Co-admission + nurse support around parenting. Liaison with outside agencies. (5–40 weeks)	Comparative	Parents admitted to the Cassell Hospital with their children. Diagnoses included personality disorder, affective disorder. Child ages: under 5 years (30 of 44).	28		Change in family functioning.	Beneficial change was identified in 14 of the families reported descriptively	3
Lenz (2004) Germany	Intervention: co-admission to mother–child ward (1–12 weeks).Control: admission without child	RCT	Mothers with children aged under 4 years. Diagnoses: depression, psychosis, personality disorder, substance misuse, OCD, mania. Child ages: mean 2.33 years (s.d. = 2.26). 25 of 44 children aged under 2 years; 19 of 44 aged over 2 years.	22 (22)	6 months to 2 years after discharge	Satisfaction with treatment experience	Intervention group significantly higher level of treatment satisfaction	2
Tritt (2004) Germany	Intervention: co-admission (6 weeks) Control: admission without child	RCT	Mothers with generalised anxiety disorder with 1–2 children. Child ages: 5–12 years	16 (17)		Parent psychiatric symptoms, changes in experience and behaviour	No significant differences	5
Verbeek (2004) Germany	Co-admission + psychoeducation on children's behaviour, feedback on parenting behaviour and interaction (using video); role play. (3.5 weeks)	Case series	Depressed mother with child aged 2 years	1	6 months and 1 year post-treatment	Parent: depression, psychosocial stress. Child: behaviour, development. Parent–child interaction and relationship	Improvement in maternal depression, improvement in child sleep behaviour, social interaction and reduced temper tantrums, separation anxiety, impulsivity, disobedience	1
Volkert (2019) Germany	Co-admission + manualised eight-session programme: three individual sessions including exploration of attachment experiences and parent–child interaction; four weekly mentalisation-focused parenting group sessions including relationship repair and how to handle difficult situations; one session on provision of social support (4 weeks)	Pre–post	Mothers with a child aged between 0 and 14 years	5		Parental stress, parental self-efficacy, participant satisfaction	Significant reduction in stress and increase in self-efficacy	1

MMAT, Mixed Methods Appraisal Tool; OCD, obsessive–compulsive disorder; RCT, randomised controlled trial.

### Intervention data synthesis

Owing to the small number of quantitative studies, heterogenous outcome measures and lack of reported effect sizes, a meta-analysis was inappropriate. Descriptive results are presented below.

### Qualitative data synthesis

Qualitative data were subject to thematic synthesis employing a three-step approach.^29^ In stages one and two, data were line-by-line coded by two raters. Codes were created iteratively and inductively and revised to generate a hierarchical code-set. The order by which papers were coded was shaped by the results of the quality assessment, so high-quality papers (those scoring >17 on CASP) were used to develop codes, and lower-quality papers were then incorporated.^28^ The text contained under each code was examined to check consistency. The third stage, carried out by A.D. in discussion with the team, generated analytic themes, informed by the research question, using an iterative process of refinement. Data forms, extracted data and data used for analyses are available from A.D.

## Results

### Study characteristics

A total of 20 551 titles were initially identified, with a further 19 from citation-chaining. Following de-duplication, 12 177 abstracts were screened, of which 64 progressed to full-text assessment. Subsequently, 24 papers met the inclusion criteria and were retained for data extraction. Of these, 16 focused on parents’ experiences, and eight were intervention studies. Eighteen studies were English-language and six were German-language (all intervention studies). See Fig. 1. Results are reported independently for the two parts of the study.

### Interventions

Eight intervention papers met the criteria for inclusion (Table 2). Seven were published in Germany (six German-language, one English-language). One was published in the UK in English. Four included an all-female sample, and the remaining four included mothers and fathers. The methodological quality of papers varied, with four achieving >80% MMAT criteria but two achieving only 20%.

### Study design and outcomes

Two papers reported RCTs.^30,31^ In both, the intervention was co-admission of mother and child, and the control group was parents admitted without children.^30,31^ Four studies employed a within-group pre–post or pre–post–post design.^32–35^

### Participants

Aggregating data from the eight intervention papers generated a sample of 428 participants. Demographic information was variable across papers (Table 3).

**Table 3:** Participant characteristics of included intervention studies. Includes data on participants in intervention group, and intervention group and control group where control is parent–child admission

	Number of papers (total)	*N*	Percentage
Gender	7 (381)		
Female		361	94.68
Male		20	5.32
Diagnosis	6 (374)^a^		
Neurasthenia		202	54.01
Affective disorders		138	36.90
Schizophrenic and psychotic disorders		9	2.41
Bipolar disorder		3	0.82
Disorder non-specified		1	0.27
Anxiety disorders		16	4.28
Personality disorders		2	0.53
Substance misuse disorders		3	0.80
Delusional disorders			
Is patient primary carer?	6 (408)		
Yes		393	96.32
No		15	3.68
Unknown			
Marital status	5 (365)^a^		
Single/divorced/widowed		253	69.32
Married/co-habiting		112	30.68

a. Includes only data on participants included in follow-up assessment in Fritz (2018).

### Summary of interventions

In all studies, the intervention was the co-admission of parent and child. We found no studies where the active intervention involved parents being admitted alone. In two studies, co-admission was the sole described intervention;^30,31^ it was the treatment-as-usual condition in two further studies.^33,35^

In six studies, parent–child co-admission was supplemented with work on parenting or the parent–child relationship.^32–37^ In one study, this comprised activities to promote parent–child interaction (e.g. movement therapy).^32^ In the single UK-based paper, parent–child co-admission was supplemented by provision of a family nurse who supported parenting and liaison with external agencies.^36^ The Leuchtturm–Elternprogramms comprised a 4-week mentalisation-oriented course, which included sessions designed to explore the parent's attachment experience, to foster positive attachment, including through relationship-repair and management of difficult situations.^37^ Volkert and colleagues piloted a mentalisation-based intervention which incorporated individual and group psychoeducation and skills training.^34^

Two papers evaluated the SEEK intervention.^33,35^ This 5-week group-based programme comprised stress reduction, psychoeducation focused on children's needs, mental health reciprocity, and increasing sensitivity to the impact of parental mental health on the child. In these papers, treatment-as-usual comprised parent–child co-admission.

In two papers, children also received psychological treatment.^36,37^

### Intervention effectiveness

#### Co-admission

##### Parent outcomes

In two studies, parent–child co-admission was associated with significant improvements in parental distress and mental health symptoms compared with baseline, with results stable at 6 months.^33,35^ Parents in one study reported significantly higher levels of satisfaction with treatment following co-admission compared with parents admitted alone.^31^ However, one paper reported no significant differences in symptoms compared with a group admitted alone.^30^

##### Child outcomes

In two studies, there was a significant post-intervention improvement in child behaviour (hyperactivity, distractibility, adaptability) but no significant improvement in behavioural or emotional problems as identified by the Child Behavior Checklist (CBCL).^33,35^

#### Co-admission plus further intervention

##### Parent outcomes

One intervention was associated with significant pre–post improvements in parent symptoms and quality of life.^32^ Another was associated with improvement in maternal depression in a case series.^37^ Two SEEK trials showed significant main effects in a pre–post design, though no within-group effects were reported. The first trial reported significant reductions in parental strain and mental health symptoms and in depression and anxiety.^38^ These were replicated in the second trial, with a significant improvement in parental strain and overall mental health symptoms after 6 months. The Lighthouse Parenting Program was associated with significant improvement in parental stress.^34^ The intervention of Healy and colleagues was associated with a descriptive account of improvement in family functioning, but no statistical analysis was reported.^36^

##### Child outcomes

There were significant improvements in children's behavioural and emotional symptoms and quality of life associated with the intervention of Besier and colleagues.^32^ SEEK was associated with significant reductions in child internalising and externalising symptoms and in behavioural and emotional problems.^33^ Effects were maintained at 6 months for overall behavioural and emotional symptoms but not for internalising and externalising subscales.^35^ In the case-series study, co-admission was associated with clinician reports of improved child sleep, social interaction, separation anxiety, and reduced temper tantrums and impulsivity compared with children who were not co-admitted with parents.^37^

## Parents’ experiences of in-patient care

Sixteen papers were included, all published in English (described in Table 4). Papers were published in the following countries: UK (five papers), USA (three), Denmark (two) Canada (one), South Africa (one), Australia (two), Greece (one) and Norway (one). Fourteen recruited from in-patient settings or in-patient settings plus community settings, and two recruited exclusively from community mental health services.^5,39^ Ten papers reported exclusively on mothers, one only on fathers and four on mothers and fathers. One study used focus groups; the remainder used individual interviews. Where stated, the analysis was descriptive (five papers), thematic (four), discourse (one), interpretative phenomenological analysis (one), grounded theory (one) and hermeneutics (one). Seven papers were identified as of low quality according to the CASP checklist. See Table 4.

**Table 4:** Description of included qualitative papers

Author (year)	Country	Participants	Methodology and data collection	Analysis	Key findings related to parenthood and care	Quality assessment (MMAT total score)
Bassett (1999)	Australia	Mothers with children under 5 years old. Sample size not reported	Qualitative using semi-structured interviews	Thematic	(a) Fear of losing residence; (b) trauma of hospital admission; (c) social isolation; (d) care of the child if mother becomes ill; (e) accessing community resources; (f) stigma of mental illness; (g) dissatisfaction with mental health services; (h) importance of relationship with children	14
Benders-Hadi (2013)	USA	24 mothers. Diagnoses: schizoaffective disorder, schizophrenia, affective disorder	Mixed methods including focus groups with semi-structured interviews	Not reported	(a) Importance of parenting role; (b) stigma; (c) difficulty of prolonged hospital stay	12
Blegen (2016)	Norway	Ten mothers with a child aged under 18 years. Diagnoses: depression, anxiety, bipolar disorder; ADHD	Semi-structured interview with philosophical hermeneutics	Hermeneutical dialectics	(a) Dare I say it? The anxiety experienced by mothers about disclosing their inner world; (b) living between the silent mask and the beating heart; the struggle between responsibility inherent in being a parent and the fear of condemnation	17
Castleberry (1988)	USA	20 patients with children aged up to 12 years admitted to in-patient hospital. Diagnoses: affective disorder, thought disorder, substance misuse disorder	Qualitative methodology incorporating whole-family perspective interviews	None reported	(a) Family life in the period coming up to hospital admission; (b) children's reactions to the separation, with particular focus on sleep; (c) how parents explained illness/hospital admission to children; (d) hospital visits. (e) partner–child relationship during hospital stay; (f) how family life adapted during hospital stay	12
Cunningham (2000)	UK	29 mothers with children aged 2–11 years. Diagnoses: depressive disorder, bipolar disorder, schizophrenia and other psychotic disorders, anxiety disorder, substance misuse disorder	Longitudinal design incorporating semi-structured interviews	None reported	(a) Children's knowledge of parental mental health; (b) feelings towards children; (c) experiences/issues since discharge; (d) support since discharge; (e) concerns for their health and their children; (f) service improvements; (g) attitude to visitation	12
Diaz-Caneja (2004)	UK	22 mothers with a child aged under 16 years old. Diagnoses: schizophrenia, bipolar affective disorder: severe depression with psychotic symptoms	Qualitative semi-structured interview	Thematic analysis using coding framework methodology	(a) Positive aspects of motherhood; (b) difficulties associated with motherhood; (c) effect of mental illness on children; (d) stigma; (e) views about services, which included (i) custody loss, (ii) adult mental health services and (iii) other agencies; (f) arrangements when mothers were unable to look after their children; (g) service needs	17
Evenson (2009)	UK	Ten fathers. Diagnoses: psychosis, schizophrenia, schizoaffective or other psychotic disorders	IPA. Semi-structured interviews	IPA	(a) Psychosis undermines the father–child relationship and the work of parenting: (i) emotional disengagement from one's children, (ii) hospital as a family disruption; (iii) medication as a straitjacket, and (iv) a negative impact on one's memory; (b) the impact of parenting on the fathers themselves: (i) pride in the father role, (ii) sense of purpose and meaning, (iii) support and understanding from children, (iv) motivation to make positive changes to one's life, and (v) fatherhood can exacerbate one's psychosis	17
Hawes (1999)	UK	26 women with a child under 16 years old. Diagnoses: psychosis or unstated	Descriptive and qualitative. Short semi-structured interviews	Descriptive report	Contact with children	12
Johnson (2009)	UK	50 mothers. Diagnoses: psychosis or unstated	Qualitative comparative	Thematic	(a) Safety and fear of other patients; (b) benefits of the company of other patients; (c) effects of the crisis house and hospital environments; (d) the stigma of admission; (e) contact with staff and opportunities to talk; (f) involvement in care; (g) women with children; (h) other treatments and activities; (i) management of medication; (j) assessment and admission; (k) the position of Drayton Park in the spectrum of local services	15
Montgomery (2006)	Canada	20 mothers with a child aged between 2 and 16 years old. Diagnoses: schizophrenia, bipolar disorder, major depression	Qualitative: grounded theory method. Unstructured interviews with informal follow-up conversations	Grounded theory	(a) Appearing normal; (b) creating security; (c) being responsible; (d) keeping close: (i) masking, (ii) censoring speech, (iii) doing motherwork, (iv) seeking help	16
Mowbray (2001)	USA	379 mothers with responsibility for at least one child aged 4–16 years old. Diagnoses: schizophrenia, schizoaffective disorder, major depressive disorders, bipolar disorders	Questionnaire-based survey with exploratory open-ended questions	Descriptive	Changes brought by motherhood	14
O'Brien (2011)	Australia	Five parents with a child aged under 18 years	Qualitative, interpretive framework	Thematic	(a) Making the decision about children visiting; (b) being responsible for the children while on the unit; (c) being a child visiting; (d) looking for help; (e) being family-friendly	18
Rampou (2015)	South Africa	Ten mothers. Diagnoses: schizophrenia, bipolar disorder, depressive disorder	In-depth interview. Explorative, descriptive and qualitative research design	Descriptive	(a) Challenges for mothers with regard to caring for their children: (i) insufficient financial resources, (ii) psychiatric symptomatology, (iii) effects of medication, (iv) lack of alternative childcare when accessing health services, (v) fear and distress about separation, and (vi) stigmatisation; (b) family support needs: (i) enhanced understanding of mental illness and treatment by mothers and their children, (ii) enhanced parenting skills, and (iii) parent-friendly arrangements for mothers who are admitted to hospital	17
Savvidou (2003)	Greece	20 mothers with a child under 18 years old Disorders: schizophrenia, delusional disorder, bipolar disorder, major depression, borderline personality disorder	Qualitative interview-based	Content analysis and discourse analysis	(a) The discourse of ‘parenthood’; (b) the discourse of ‘mental illness’ and ‘mentally ill parent’	13
Wang (1994)	Denmark	50 parents or stepparents of children aged 0–10 years. Diagnoses: mental and behavioural disorders due to psychoactive substance use, schizophrenia, schizotypal and delusional disorder, neurotic, stress-related and somatoform disorders, disorders of adult personality and behaviour	Cross-sectional, descriptive, and based on semi-structured interview.	Data categorised and reported descriptively	(a) Concern over child development and health; (b) relationship with children; (c) view of own mental health; (d) cooperation with professionals	13
Wang (1996)	Denmark	50 parents or stepparents of children aged 0–10 years. Diagnoses: mental and behavioural disorders due to psychoactive substance use, schizophrenia, schizotypal and delusional disorder, neurotic, stress-related and somatoform disorders, disorders of adult personality and behaviour	Cross-sectional, descriptive, and based on semi-structured interview	Data categorised and reported descriptively	(a) Professional help relating to children; (b) additional needs for help; (c) establishing contact with professionals; (d) children's extrafamilial contacts; (e) children at time of hospital admission; (f) advice from parents to professionals	13

ADHD, attention-deficit hyperactivity disorder; IPA, interpretative phenomenological analysis.

### Participants

Participant data from 14 papers were aggregated (one paper failed to report sample size,^40^ and one was excluded because the study employed a sample that had been reported elsewhere and already included in the count^41^ to generate a combined sample of 629 participants. Gender, diagnoses care-giving responsibilities and marital status of participants are reported in Table 5.

**Table 5:** Participant characteristics of in-patient parent ‘experience’ studies

	Number of papers reporting (total)	*N*	Percentage
Gender	11 (584)		
Female		577	95.53
Male		27	4.47
Diagnosis	12 (615)		
Affective disorders		224	36.42
Schizophrenic and psychotic disorders		158	25.69
Bipolar disorder		121	19.67
Disorder non-specified		78	12.68
Anxiety disorders		13	2.11
Personality disorders		10	1.63
Substance misuse disorders		10	1.63
Delusional disorders		1	0.16
Is patient child's primary carer?	9 (209)		
Yes		151	72.25
No		51	24.40
Unknown		7	3.35
Marital status	10 (590)		
Single/divorced/widowed		333	56.44
Married/co-habiting		256	43.39
Unknown		1	0.17

### Key themes

Parents’ experiences were categorised into six themes: ‘who is looking after my child?’; ‘maintaining connection from hospital’; ‘impact on self as a parent’; ‘discharge is not the end’; ‘perceived child experience’; and ‘what needs to change’. Together, these represent the impact of hospital admission in terms of the parent's physical separation from their children as well as its effects on the parenting role, on the child, and on the parent's self-concept. The final theme integrates views on improvements that could be made to better support parents.

### Who is looking after my child?

This theme, present in seven studies, focuses on care arrangements in place for children while their parent is in hospital. Many parents expressed worry, confusion or anger about these arrangements, as described by one mother:

‘*On the ward, there were women who were very distressed by the fact that their children were in care somewhere and they couldn't see them, and I just think that it is so damaging.*’^5^

Parents identified difficulties in arranging care or having no-one to care for their children during their hospital stay, and, in cases where children were under the care of social services, not knowing who was looking after their child was a source of distress:^5,42^

‘*When I was admitted at the hospital I was thinking of my children, where are they? Who is taking care of them? Who is bathing and making food for them?*’^42^

Even when co-parents or family members were looking after children, it was a source of worry or ambivalence.^43^ The exception was one study where four parents said they felt their children were looked after well.^44^

A number of parents associated hospital admission with a risk of permanent removal of children.^5,40,42,43,45,46^ The threat of child-removal was a source of extreme distress in all accounts. Some parents reported worrying that the alternative care arrangements during their hospital stay made the subsequent removal of their child more likely, whether into the care of a co-parent as in the example below or into the care of the state.^40,42,43^

‘*I'm the one who fears that he [father] might take them from me saying that I'm an unfit mother.’*

Another stated that:

*‘I was scared when I was admitted at the hospital that they would take my children from me. I don't want them to stay with somebody or be taken away from me … ’*     ^42^

Parents who had experienced removal of a child characterised it as highly distressing and detrimental to their recovery.^5,42,45^

#### Maintaining connection from hospital

Ten studies featured parental accounts that discussed maintaining connection. For most, hospital admission involved a physical separation from their children, which was accompanied by a desire to maintain some form of connection, for example, through regular telephone contact.^5,39,40,47–49^

Child visitation was mentioned in several studies.^5,39,47,48,50,51^ However, visits were ambivalently represented. In two papers, children were described as making regular visits to parents, including eating meals with them.^47,48^ However, for many parents, the desire to see children conflicted with concerns about the risks of visiting, including the suitability of the ward environment, owing to the lack of appropriate visiting facilities, and about exposure to other unwell patients.^5,39,48,49,51^

‘*I'm a little cautious about having them in to see me, the ward, cause . . . some people are quite disturbed and it can be quite upsetting for them*.’^39^

Some parents did not want their child to see them while they were unwell.^39,47,49^ One father described having been admitted to hospital annually for periods in excess of a month but refusing visits from his child for this reason.^39^

For some, the distance afforded by admission offered a break from the stresses of parenting and/or family life, after which the parent was able to ‘return to bond with their children as a “new mom”.’^43,51^

#### Impact on self as parent

Nine studies explored the impact of hospital admission on parenting identity. The experience of hospital admission and the poor mental health preceding it appeared to diminish parents’ belief in their value as a parent.^39,42,44,47,52^ Hospital admission was described as preventing parents from fulfilling their parental responsibilities.^39,42,47^ It meant parents were ‘not available’,^42^ as one father described:

‘*I haven't been there for them sometimes because I've been in hospital . . . I miss out, and my son misses out on my contact*.’^39^

Some parents viewed their parenting negatively because of harms they felt they may have caused their children through exposure to their symptoms.^43,44,47,52^ For example, mothers in the study by Montgomery and colleagues felt they struggled to meet their ‘primary responsibility’, which was to protect their children from their illness.^43^ This was accompanied by the fear that they may have ‘inadvertently hurt’ their children. Rampou and colleagues describe parental concern about children being cast into a parental role by their parent's illness, in particular where a child took on caring responsibilities.^42^

Parents also described the corrosive effect of stigma associated with hospital admission.^1,39,40,42,44–47^ For some, this was related to shame about being unwell and admitted to hospital; this self-stigma was related to their view of their own illness or the hospital environment:

*‘inpatients in psychiatric units are mad and dangerous’.*^45^

Others felt that hospitalisation affected the way they were perceived and treated by others, including the access they were given to their children:^1,39,40,51^

‘*And then it was treated like it was something to be ashamed of and I think that's why society's attitude that mental illness is something that it's sort of like having ‘crazy bitch’ stamped across my forehead and everybody treats you differently because you have been a patient in a psychiatric unit*.’^40^

For some, this stigma, rather than illness or hospital admission, was represented as the thing which had most affected them as parents,^1^ as one mother expressed:

‘*I'm probably afraid of being labelled a lunatic and then they will take my children away*.’^46^

#### Discharge not the end

In six studies, parents described challenges after their hospital stay. Although returning to the parental role motivated engagement with treatment for several parents,^1,5,45^ discharge was not always viewed as straightforwardly positive.^1,5,40,44,51^ No paper explicitly discussed the threshold at which parents had been discharged; however, there were clear indications that many were still unwell and worried about their ability to cope with the practicalities of parenting.^39,42,44,51^ In one example, a father found the intensity of family life too great and returned to hospital.

‘*Four young children . . . all under 5 and that, and they're flying about, large as life all the time. You know, as soon as I got home, after coming out of a quiet hospital, you know it was too much for me. I had to go back in*.’^39^

The failure of in-patient settings to engage with the parenting role was felt to contribute to these difficulties.^43,46^ By focusing on symptoms and failing to give attention to the parental role and the specific needs of parents, treatment failed to support them in the resumption of that role:

‘*Since I've been in treatment I'm not anxious, I'm thinking better, I'm sleeping, I can focus but what about when I leave to go home? I will keep seeing [the psychiatrist] but it is the other stuff that worries me … when I have to get up at night, when I have to play with them but I'm tired*.’

An additional complication of discharge related to difficulties in re-establishing relationships with children.^1,5,44,51^ One aspect focused on the child's understanding of the separation and feelings of rejection.^5^ Other parents highlighted their inability to communicate their experiences of hospital admission or illness or to explain their absence.^1,5,47^ One mother pulled these strands together when describing aspects of parenting that she felt she would have to re-learn:

‘*It's like I have to learn how to be around my kids again … how to get along with them, how to tell them I love them, and how to explain that I wasn't there because I'm sick*.’^1^

The potential of readmission was raised by some studies. For some parents, preventing future hospital admission motivated treatment adherence,^53^ whereas in other accounts, help-seeking was avoided owing to the risk of readmission and the accompanying separation from children.^5,41^ Just as some parents experienced hospital admission as a ‘hostile’ act,^43,45^ another expressed fear that the father of her children would use it as an aggressive act towards her:

‘*I'm scared. I'm scared. I'm so scared*.’^40^

#### Perceived child experience

Parents’ thoughts about their children's experiences was reported by eight studies, though in limited form.^39,42,44,45,47^ In some cases, hospital admission of the parent was described as having a negative impact on the child's affect or behaviour:^42,44,45,47^

‘*It looks like he's holding something, a worry inside. When he came to the hospital the next day to see me, he was so quiet and bashful. I could hardly get him to talk.*’

However, other parents described their children as adapting to or having ‘an understanding’ of the situation.^44,47^

As for earlier themes, where parents discussed the impact of their hospital admission or health on their children, it was frequently associated with feelings of shame, and the focus of discussions was often on self-blame rather than the experience of the child.

#### What needs to change?

Nine studies highlighted a need for improved provision to better enable parents to maintain their parenting role while undergoing in-patient treatment. The clearest target for improvement was the development of appropriate facilities for children to visit parents during treatment.^5,42,44,49^ This included ‘family rooms’ or private spaces away from the main ward:^5,44^ ‘somewhere quieter’^51^ and ‘away from other patients’.^5^ In some studies, there was a call for co-admission of child and parent.^5,42^

The second key suggestion was that parental identity should be engaged with and supported^41,42,46,53^ and that staff should engage with this with openness, persistence and empathy – in the words of one parent, ‘to show more love’.^41^ As described by one parent, therapists need to enable parents to share what they hold as important:

‘*I feel I have much at heart, but when I arrive he asks me about how I have been since the last time, and continues with that, including techniques and exercises, and I have no opportunity to say what I was going to say*.’^46^

Parents also proposed that greater effort should be made to identify that a patient is a parent^44^ and that support should aim to strengthen parental functioning, promote parenting skills and ease transition home.^41,42,52,53^

## Discussion

The studies that explored the experiences of in-patient parents indicated that parents largely experience psychiatric in-patient care negatively and find that it has an impact on their ability to function in their parenting role. This impact arises as a consequence of several factors. Chief among these is the physical separation of parents and children, but the impact of stigma (self and external) was also clear. Parents’ concern about their ability to provide care once discharged and worry about the potential loss of their children also featured widely.

Where parents described what improvements should be made, the inappropriateness of facilities for child visits was emphasised. It is noteworthy that poor provision of visiting facilities was highlighted in the oldest included paper and continued to be flagged as a concern by parents 25 years later. This echoes a 2021 review where women identified a similar tension between the negative impact of separation and belief that hospital was unsafe for child visits.^14^ The lack of appropriate provision for children's visits has also been identified as a concern by psychiatric nurses and by children themselves.^50,54^

However, although there has been some research interest in the experiences of parents who are in-patients, there has been comparatively little work attempting to develop or evaluate interventions for this group. Even employing broad search criteria, the results of this review include just a handful of interventions. Moreover, all of these studies examined an intervention that was centred on co-admission of parent and child, and all but one reported interventions that were delivered in the German health service. Co-admission was largely associated with positive outcomes for parents and children and with high treatment satisfaction. In Germany, co-admission exists within the broader health system through a network of ‘Mutter-Kind-Einrichtungen’ in which parents receive care alongside their child. However, these centres are not psychiatric wards; rather, they provide rehabilitative and preventive holistic treatment to parents who experience psychological and/or physical ‘exhaustion’.^55^ These settings reflect a wider, systemic engagement with the parent *and c*hild in addressing parental difficulties. By contrast, only one intervention study from elsewhere in the world (UK) was identified. This involved co-admission of parent and child, but this provision is now no longer offered.^36^ Although co-admission may not be a realistic ambition within the UK mental health system, supporting contact between parent and child is realistic and should clearly be prioritised.

Studies that offered parent-oriented interventions in addition to co-admission provided tentative evidence of effectiveness. These interventions, ranging from supporting parent–child interaction to structured multi-session psychoeducational interventions, were associated with improved outcomes for parent, child or parenting, or a combination of these.^21,56^

### Clinical implications

The results of this review suggest three areas where improvements could have substantial impacts on in-patient parents and their children.

### Family-friendly visiting rooms

In the UK, the Mental Health Act (1983) states that every effort should be made to support in-patient parents to maintain contact with relatives and to continue to support their children. It is clear that, in order to facilitate this, family-friendly spaces should be available in all psychiatric in-patient settings, and these, if managed well (ensuring privacy and safety), would be welcomed by parents. Unfortunately, a recent review by the Scottish Executive highlighted ‘patchy’ provision of child-friendly visiting spaces,^57^ and the situation is likely to be similar across the UK. In Australia, implementation of family-friendly rooms has been associated with cascading benefits, from maintenance of the parent–child relationship to promotion of parental recovery. Staff have also suggested that these spaces may be associated with a reduction in stigma experienced by parents.^58^ In creating an appropriate environment for children, family-friendly spaces could also address the negative attitudes and experiences children associate with their parent's mental health.^16^

### ‘Patient as parent’ thinking

It is clear that in-patient units could engage better with their patients’ identities as parents. Such ‘patient as parent’ thinking could include improved recognition of the parenting role in general ward care (e.g. asking about patients’ families, encouraging them to talk about their children and their concerns for them). In addition, it is likely that interventions that support parenting and, in particular, the parent–child relationship, would have benefits for both parent and child upon discharge. Currently, such engagement is *ad hoc* and uncommon: a recent survey of British mental health workers found that in-patient staff were the least likely of any professional group to engage with patients in terms of their parenting role.^20^ The willingness of staff to engage in this way is influenced by a range of factors including confidence and training.^20^ However, given that both staff and parents recognise the importance of validating a patient's parenting role, there is a need for services to do so.^20,59^ Furthermore, tentative evidence suggests that when staff do engage with their clients’ parenting role, there is potential for cascading benefits for the parent–child dyad.^60^

### Parent and child co-admission

Several studies identified parent–child co-admission as a suitable method for supporting the parent–child relationship during hospital treatment. It was viewed positively by most parents who experienced it, although a minority described it as a potential impediment to treatment effectiveness. However, this approach is likely to be considerably more expensive than solitary admission of parents, and major changes to service infrastructure would be required.

### Strengths and limitations

This review employed purposely broad inclusion criteria yet found only a small number of reports of interventions delivered to parents accessing in-patient care. As such, it may not represent the full range of experiences. Furthermore, the search strategy did not include grey literature, which may have excluded reports of small-scale interventions.

Strengths included the use of thorough and inclusive search terms and accessing of papers written in two languages (German and English) which, between them, are likely to capture a large amount of the literature. However, the failure to include papers in additional languages may have biased results towards research carried out in high-income countries. This limits exploration of the experience and support for in-patients in a wider range of health systems. High levels of heterogeneity in the data rendered meta-analysis inappropriate.

In drawing together literature from a range of nations, the review sought to engage with experiences of in-patient parenthood. Although the clear identification of common experiences and needs is a strength, there would be utility in engaging with the impacts of different health systems and cultural norms, which may affect the delivery and experience of care. Furthermore, individual, familial and treatment variables are likely to affect a parent's experience of care. Although there was not opportunity within this review to engage with these factors, they warrant further investigation.

Given the limited data, the variation in extent and form of engagement, and the reliance on within-groups analysis (including when a control group was present), the results of the current review should be interpreted cautiously. However, although this evidence gap is disappointing, it demonstrates the need for research in which interventions are scrutinised. Furthermore, in designing studies, researchers should incorporate standard outcomes relating to parent and child well-being, as well as the specific behavioural or functional targets of the programme.

### Implications for in-patient provision

Bringing together the evidence on the parental experience of in-patient care and the provision offered to in-patient parents makes the unmet need within services clear. Hospital admission of a parent typically reflects a situation in which the parent's mental health precludes them from caring for their child. This may exist alongside other adversity such as socioeconomic disadvantage, lack of social and familial networks, housing instability, and interparental conflict or abuse.^61–63^ Whereas in-patient care cannot address the multiple vulnerabilities faced by some parents, it should not contribute to them by hindering the relationship between parent and child. Although this review is unable to provide recommendations on the form and content of future interventions, it can conclude that, at the very least, in-patient provision should identify and engage with the parenting identity of parents and offer appropriate facilities for patients’ children to visit.

## Data Availability

This study is a re-analysis of existing data which are openly available at locations cited in the ‘References’ section of this paper.

## Supplementary material

For supplementary material accompanying this paper visit http://doi.org/10.1192/bjo.2023.67. click here to view supplementary material
