# Supplementary material for: Psychiatric in-patients who are parents: what interventions are tailored to their needs and how do they experience care? A systematic review and data synthesis
Source: BJPsych Open. 2023 Jun 22;9(4):e111. doi: 10.1192/bjo.2023.67 (PMC10305023; doi:10.1192/bjo.2023.67)
Supplement: Supplementary file 1 [file S2056472423000674sup001.docx]

**Appendix A**

| Bibliographic details  Author(s)  Date  Linked studies | |
| --- | --- |
| **Intervention studies** | **Qualitative studies** |
| - Study aim and objectives - Research question - Design - Setting - Recruitment methods - Consent - Inclusion/exclusion - Number of participants - Participant characteristics (as appropriate: age, gender, ethnicity, diagnostic information, service characteristics - Intervention(s) characteristics (including description, frequency, delivery) - Comparator (if appropriate) - Study outcomes (primary and other) - Number of participants included in analysis - Follow-up - Reported intervention effects - Funding and sponsorship | - Study aim and objectives - Research question - Theoretical/epistemological perspective - Design - Setting - Recruitment - Consent - Inclusion/exclusion - Number of participants - Participant characteristics (as appropriate: age, gender, ethnicity, diagnostic information, service characteristics - Data collection - Analysis - Reflexivity - Outcomes - Findings: Themes - Conclusion - Funding and sponsorship |
